# Spontaneous Combustion of Pulverised Charcoal

**Published:** 1831

**Authors:** 


4. *Spontaneous Combustion of Pulverized Charcoal.*—The occurrence of spontaneous combustion in finely-pulverized charcoal, was so frequent, that a series of experiments seemed to be peculiarly called for to determine the cause. These experiments were made at one of the Polytechnic schools of France, in 1828, and the result is as follows:

Charcoal in an open vessel, undergoes spontaneous combustion, while a portion of the same kind of charcoal, of equal weight, in a perfectly tight vessel, remains unchanged; and the inference is, that the absorption of atmospherical oxygen is essential to the combustion.—*Annales de Chimie.*

